# Heterotopic Pregnancy After Intracytoplasmic Sperm Injection With Viable Embryos: A Case Report and Literature Review

**DOI:** 10.7759/cureus.90540

**Published:** 2025-08-19

**Authors:** Ioannis Boutas, Adamantia Kontogeorgi, Sofia-Maria Genitsaridi, Maria Sotiria Bompoula, Nektarios I Koufopoulos, Kyparissia Sitara, Dionysios T Dimas, Sophia Kalantaridou, Antonios Makrigiannakis, Magda Zanelli, Andrea Palicelli, Maurizio Zizzo, Giuseppe Broggi, Serena Salzano, Rosario Caltabiano, Annette Hasenburg, Roxana Schwab

**Affiliations:** 1 Department of Obstetrics and Gynecology, University Medical Center of the Johannes Gutenberg University Mainz, Mainz, DEU; 2 Third Department of Obstetrics and Gynecology, Attikon University Hospital, National and Kapodistrian University of Athens, Athens, GRC; 3 Department of Pediatrics and Neonatology, Agia Sofia Children's Hospital, Athens, GRC; 4 Second Department of Pathology, Medical School, Attikon University Hospital, National and Kapodistrian University of Athens, Athens, GRC; 5 Department of Internal Medicine, “Elpis” General Hospital of Athens, Athens, GRC; 6 Breast Unit, Athens Medical Center, Psychiko Clinic, Athens, GRC; 7 Third Department of Obstetrics and Gynecology, Medical School, Attikon University Hospital, National and Kapodistrian University of Athens, Athens, GRC; 8 Department of Obstetrics and Gynecology, University Hospital of Heraklion, Heraklion, GRC; 9 Pathology Unit, Azienda Unità Sanitaria Locale Istituto di Ricovero e Cura a Carattere Scientifico (USL-IRCCS) di Reggio Emilia, Reggio Emilia, ITA; 10 Surgical Oncology Unit, Azienda Unità Sanitaria Locale Istituto di Ricovero e Cura a Carattere Scientifico (USL-IRCCS) di Reggio Emilia, Reggio Emilia, ITA; 11 Department of Medical, Surgical Sciences and Advanced Technologies “GF Ingrassia” Anatomic Pathology, University of Catania, Catania, ITA

**Keywords:** ectopic pregnancy, heterotopic pregnancy, intrauterine pregnancy, in vitro fertilization, risk factors

## Abstract

Heterotopic pregnancy is defined as the simultaneous presence of gestation in two different sites, typically comprising an intrauterine pregnancy and an ectopic pregnancy. Due to the widespread use of assisted reproductive technologies (ARTs), the incidence of heterotopic pregnancies has increased significantly.

A 36-year-old Caucasian woman, gravida 1, para 0, Rhesus positive, was referred to our hospital with a suspected ectopic pregnancy following her third in vitro fertilization with intracytoplasmic sperm injection cycle. The patient reported no vaginal bleeding or abdominal pain at the time of admission. Her medical history included dysmenorrhea, dyspareunia, and prior hysteroscopic removal of an endometrial polyp. She had no other significant medical, surgical, or family history. Transvaginal ultrasound confirmed the diagnosis of a heterotopic pregnancy. The patient underwent surgical management to address the ectopic component.

The incidence of heterotopic pregnancy has risen in recent years due to the increased use of ARTs. In cases of tubal ectopic pregnancy, surgical intervention should be carefully considered, even when the risk to the coexisting intrauterine pregnancy cannot be entirely excluded.

## Introduction

A heterotopic pregnancy is defined as the simultaneous occurrence of an ectopic and an intrauterine pregnancy. This is an exceptionally rare event in natural conception cycles, with an estimated incidence of one in 30,000 pregnancies [1]. However, the incidence has significantly increased to 1%-2.1% in recent decades, primarily due to the rise in assisted reproductive technologies (ARTs) [1-3]. In in vitro fertilization/intracytoplasmic sperm injection (ICSI) and embryo transfer (ET), the general incidence of ectopic pregnancy is 2.12%, whereas heterotopic pregnancies constitute a smaller percentage, approximately 0.27% [4].

Ectopic pregnancies (EP) remain a leading cause of maternal morbidity and mortality during the first trimester [5,6]. Most ectopic pregnancies occur in the fallopian tubes, with 80% located in the ampullary portion, 15% in the isthmic portion, and 5% in the fimbrial end. This distribution is consistent regardless of whether the pregnancy occurs spontaneously or through ART [7,8].

Several studies have explored risk factors and predispositions for EP following ART. These include tubal abnormalities, blastocyst-stage ETs, endometriosis, and reduced endometrial thickness [9-11]. Endometriosis, which affects approximately 40% of infertile women, is associated with a heightened risk of EP, particularly in ART contexts [12-14]. This condition is characterized by the presence of endometrial-like tissue outside the uterine cavity [15], resulting in chronic inflammation, adhesions, and fibrosis [16].

Patients with endometriosis often present with symptoms such as chronic pelvic pain, dyspareunia, dysmenorrhea, and infertility. However, some cases are asymptomatic, making diagnosis challenging [17,18]. While ART remains the optimal treatment for endometriosis-related infertility [19-21], the risk of EP in such cases is not yet fully understood. In this report, we present a rare case of a heterotopic pregnancy following ICSI, with a viable intrauterine and ectopic pregnancy in the right fallopian tube.

## Case presentation

A 36-year-old woman, gravida 1, para 0, Rhesus positive, and of Caucasian origin, was referred to our hospital with suspected ectopic pregnancy following her third ICSI attempt. She denied experiencing vaginal bleeding or abdominal pain. Her medical history included dysmenorrhea, dyspareunia, and a hysteroscopic endometrial polyp ablation. The patient has presented with a three-year history of couple infertility and has undergone three cycles of fertility treatment with ICSI. Her past medical history was unremarkable, with no previous surgeries or known chronic comorbid conditions.

On physical examination, no signs of intra-abdominal fluid leakage or active bleeding were observed. The cervix was closed, long (4.1 cm), and posterior. Laboratory evaluation revealed a beta human chorionic gonadotropin level of 32,000 mIU/mL, consistent with a gestational age of five to six weeks. The remaining laboratory tests were within range, as shown in Table 1.

**Table 1:** Laboratory values of the patient before laparoscopy β-hCG: beta human chorionic gonadotropin; CBC: complete blood count; WBC: white blood cell; CRP: C-reactive protein; TSH: thyroid-stimulating hormone

Test	Value	Normal range
β-hCG (quantitative)	32,000 mIU/mL	Normal from fifth to sixth week of pregnancy
Progesterone	21 ng/mL	>10-25 ng/mL
CBC (hemoglobin)	12.2 g/dL	11-13.5 g/dL
Hematocrit	36%	33%-39%
WBC	12,000/μL	6,000-16,000/μL
Platelets	180,000/μL	150,000-400,000/μL
CRP	16 mg/L	<5 mg/L
Urea	12 mg/dL	7-20 mg/dL
Creatinine	0.6 mg/dL	0.4-0.8 mg/dL
TSH	2.1 μIU/mL	0.1-2.5 μIU/mL

**Figure 1:**
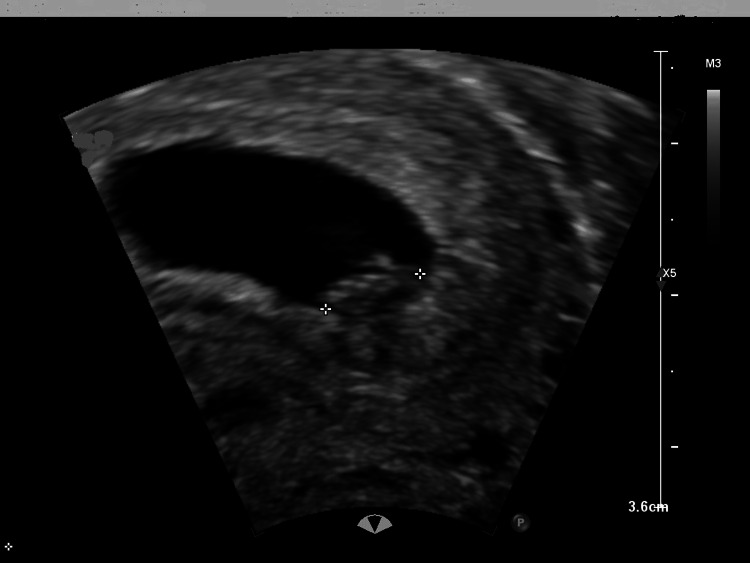
Intrauterine pregnancy showing fetal pole

**Figure 2:**
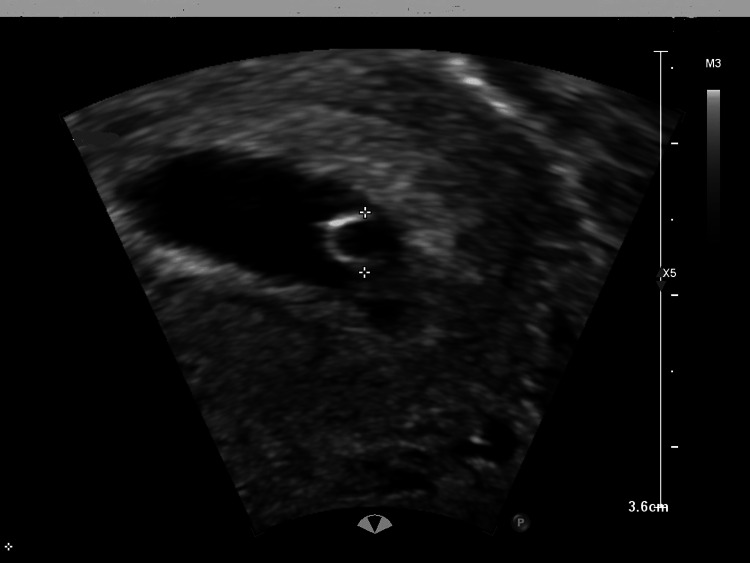
Intrauterine pregnancy showing yolk sac. In early pregnancy, the yolk sac is one of the first structures seen on ultrasound and provides important information about the development and health of the pregnancy

**Figure 3:**
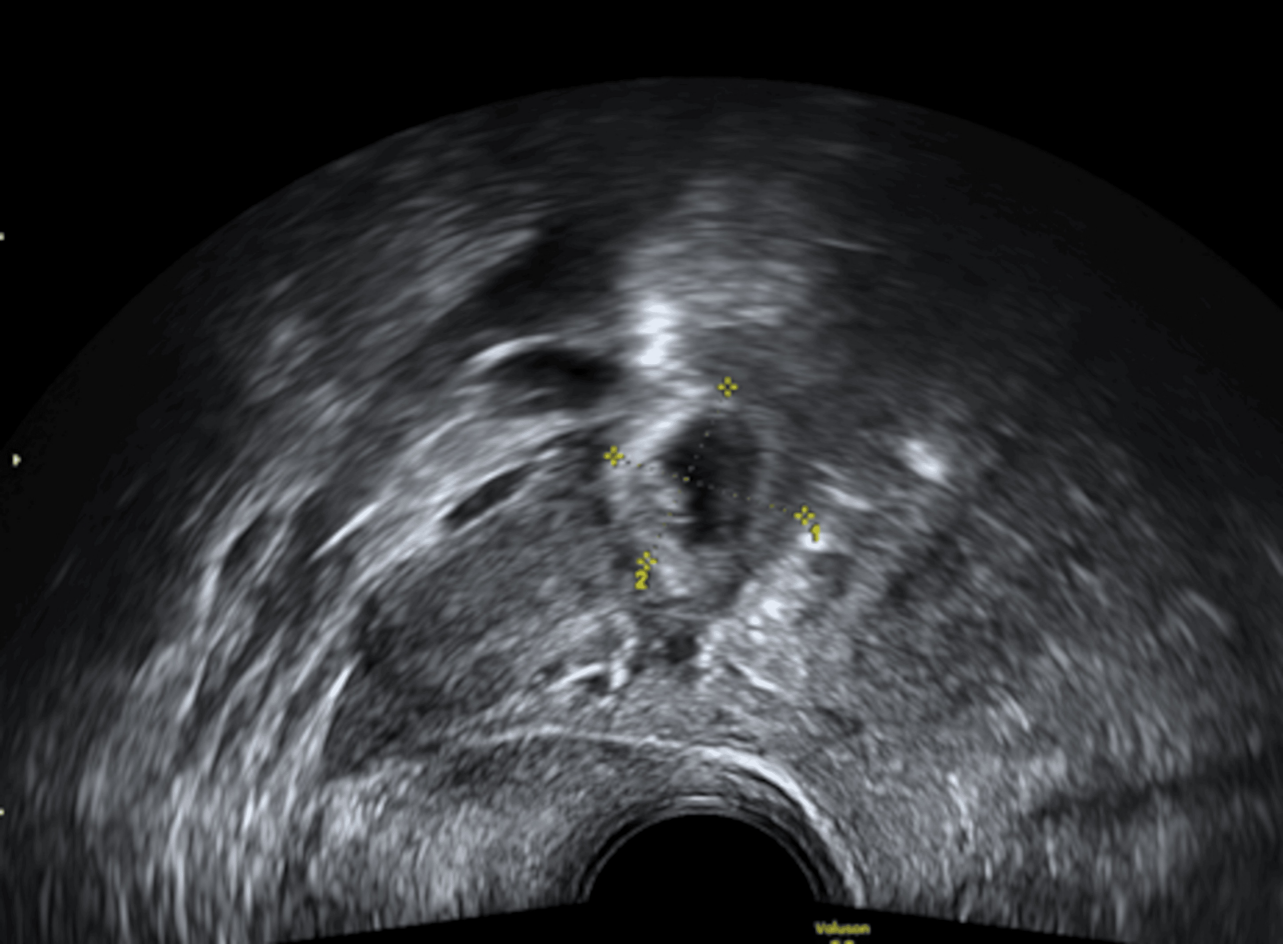
Right tubal ectopic pregnancy

**Figure 4:**
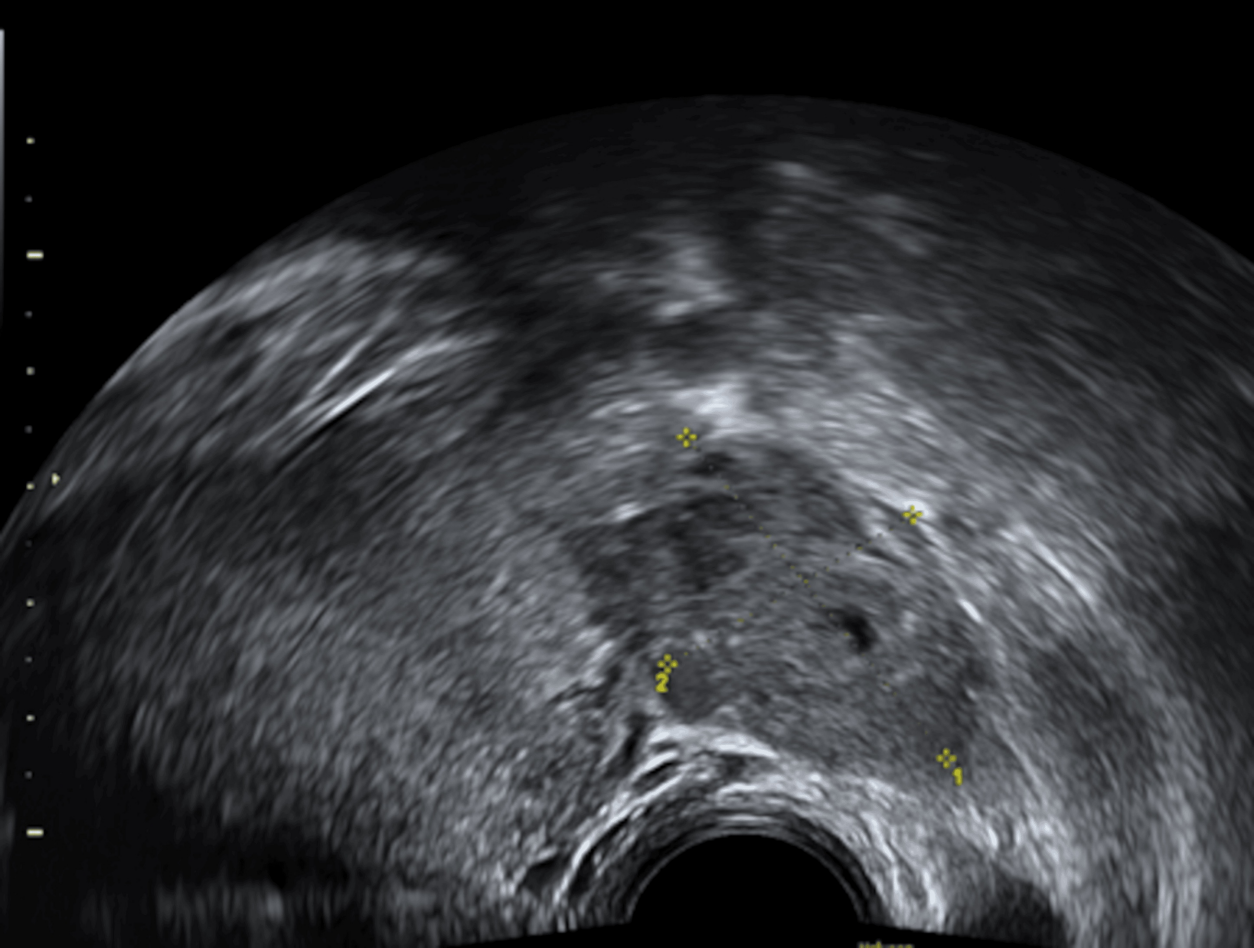
Ultrasound images suggesting ectopic pregnancy with a right ovarian mass

Transvaginal ultrasound indicates a viable intrauterine pregnancy at six weeks of gestation based on crown-rump length (Figures 1, 2), alongside a concurrent viable ectopic pregnancy in the right fallopian tube, measuring 1.73 cm (Figures 3, 4).

The diagnosis of heterotopic pregnancy was established. The patient was counseled extensively about the condition, including the potential complications of a heterotopic pregnancy, such as rupture and hemorrhage, which could endanger her life and the intrauterine pregnancy. Discussions included the importance of timely surgical intervention to remove the ectopic pregnancy and safeguard the viable intrauterine pregnancy. The potential risks of miscarriage due to the surgical procedure were clearly communicated, and the patient was provided with written and verbal information to ensure understanding. Alternative management options, including expectant management and medication, were discussed in detail. Expectant management was deemed inappropriate due to the high likelihood of tubal rupture and subsequent life-threatening complications. The patient was informed of the potential consequences of delaying treatment and was encouraged to ask questions to ensure she was fully informed about her options. The patient, after receiving thorough counseling and having all her questions addressed, provided informed consent for the surgical intervention. She expressed an understanding of the risks, benefits, and potential outcomes of the procedure.

In this case, laparoscopy was performed during the first trimester of pregnancy with consideration of maternal and fetal safety. The pneumoperitoneum was established using carbon dioxide (CO_2_), with an intra-abdominal pressure maintained at 10-12 mmHg, which is within the recommended range for pregnant patients. Lower insufflation pressures are advised in pregnancy to minimize the risk of reduced uteroplacental perfusion and fetal acidosis. The initial flow rate was set at 1-2 L/minute and gradually increased as needed, with close monitoring of the patient’s cardiopulmonary status. These settings are in line with current guidelines for minimally invasive surgery in pregnancy and provide adequate visualization while ensuring physiological stability.

**Figure 5:**
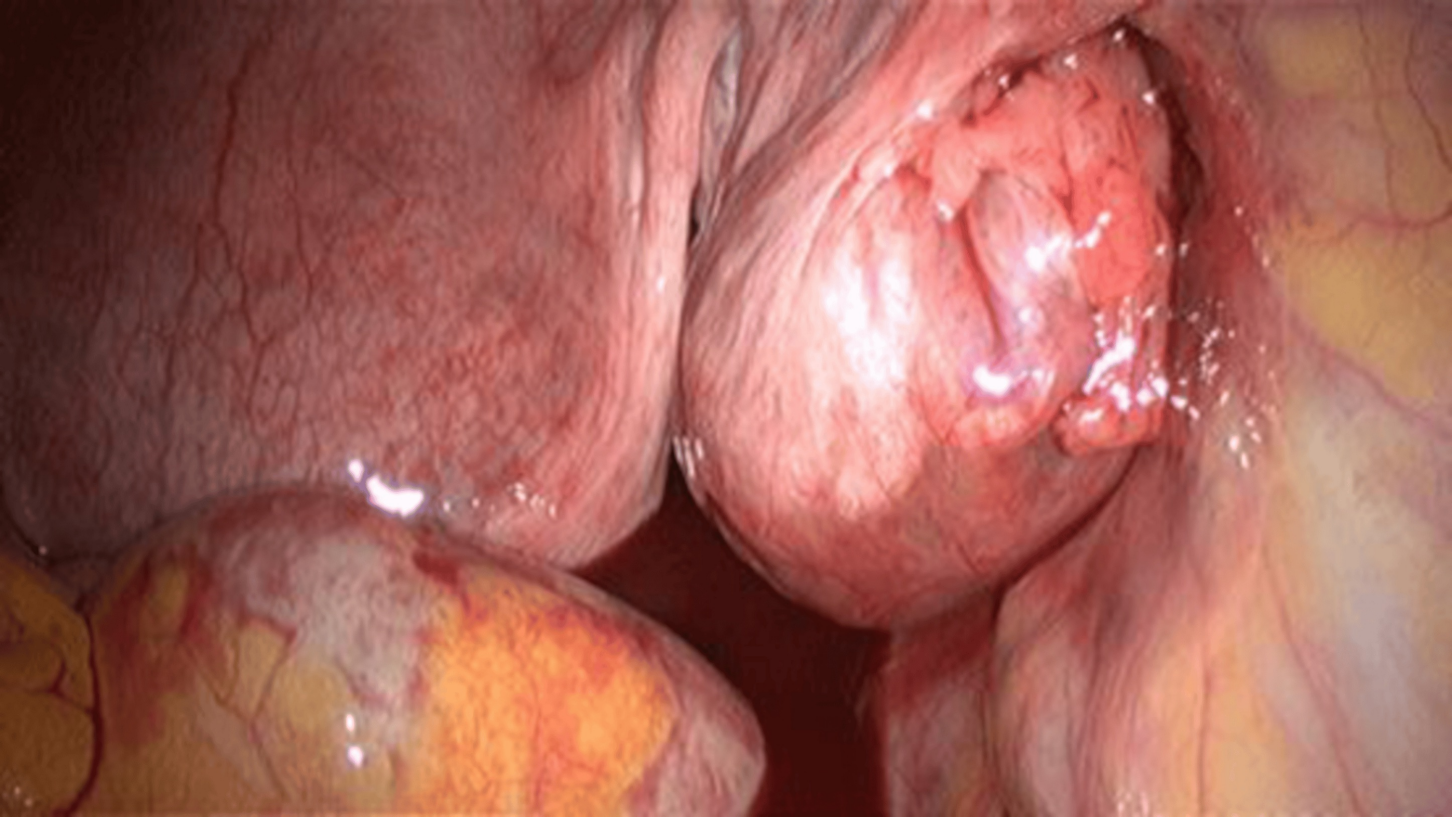
Laparoscopic findings confirming ectopic pregnancy with an ovarian mass on the right ovary

**Figure 6:**
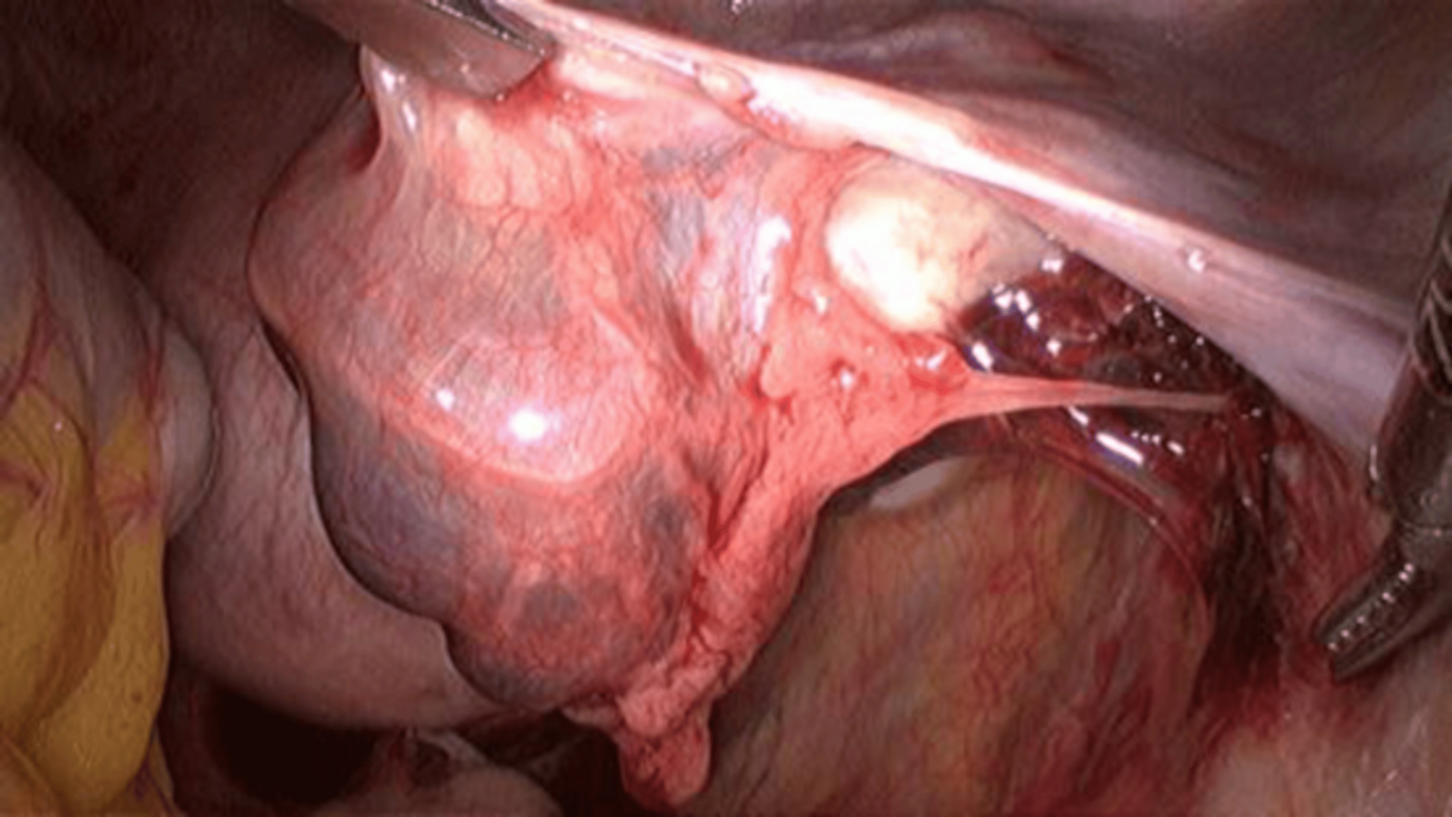
Laparoscopic findings of tubal pregnancy into the fallopian tube lumen

**Figure 7:**
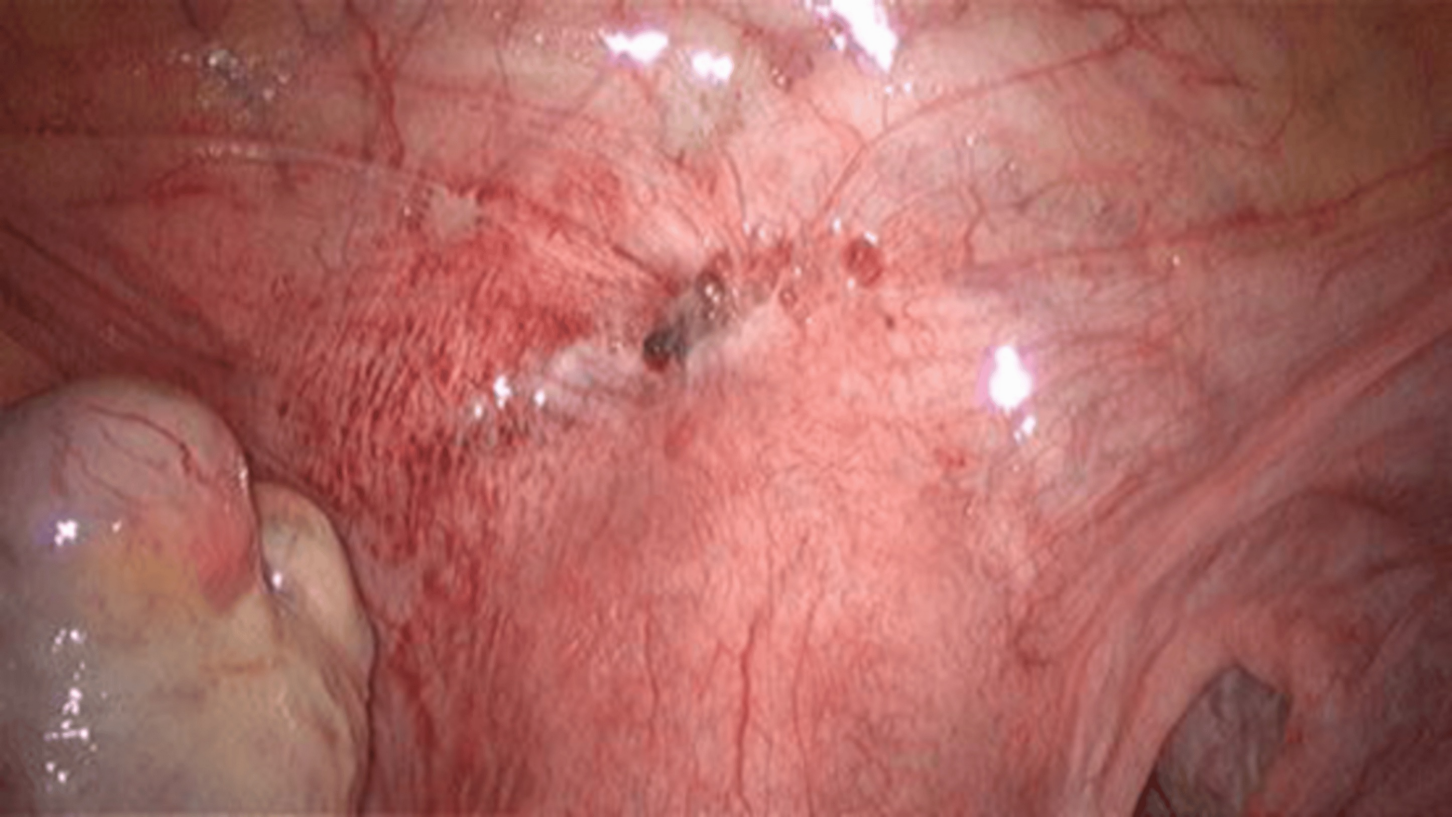
Endometriosis spots in the vesicouterine pouch

Upon entry to the abdomen, a mass in the right tubal cyst was observed, as well as endometriosis spots in the vesicouterine pouch (Figure 5). A second and third trocar were placed to facilitate visualization of the ectopic mass (Figure 6). Blunt graspers were used to elevate the uterus, and a right tubal pregnancy was identified with a subsequent salpingectomy. During laparoscopy, endometriotic lesions consistent with stage I (minimal) endometriosis were identified in the vesicouterine pouch, the space between the bladder and the uterus. This staging is based on the revised American Society for Reproductive Medicine (rASRM) classification system and reflects the presence of a few small superficial implants without significant adhesions. Biopsies were taken from the affected area to confirm the diagnosis histologically. Although minimal, such lesions in this location can still contribute to pelvic pain or infertility (Figure 7).

Postoperatively, the patient’s recovery was uneventful. It was not possible to pursue conservative pharmaceutical management, as this would endanger the viability of the intrauterine pregnancy. This was explained to the patient, and it was also advised to continue routine antenatal care.

## Discussion

This case contributes to the existing literature by highlighting the successful management of a heterotopic pregnancy in a patient with a history of endometriosis and prior ART cycles. The presence of visible endometriosis lesions during laparoscopy and the classification of rASRM Stage I added valuable insights into the potential correlation between endometriosis and the increased risk of heterotopic pregnancy. Furthermore, the successful continuation of the intrauterine pregnancy following laparoscopic intervention demonstrated the feasibility of preserving intrauterine viability in carefully managed cases.

Recent studies have reported varied outcomes for heterotopic pregnancies managed surgically [22]. A 2024 retrospective analysis by Xiao et al. involving 50 cases of heterotopic pregnancies showed that laparoscopic salpingectomy had a live birth rate of 75% resulting from the intrauterine pregnancy, aligning closely with the outcomes of this case [23]. Another 2024 study by Wang et al. emphasized the role of early ultrasound diagnosis, reporting a significant reduction in complications when heterotopic pregnancies were identified and managed within the first trimester [24]. Unlike cases with delayed diagnosis, our case illustrated the critical importance of timely intervention and patient counseling in achieving favorable outcomes.

Additionally, studies have explored the impact of ART-related risk factors. A 2023 meta-analysis by Krishnamoorthy et al. found that multiple ETs and fresh ETs significantly increased the incidence of heterotopic pregnancies, with a 40% higher risk in women undergoing controlled ovarian stimulation. These findings underscore the importance of single ET policies in reducing heterotopic pregnancy rates. The patient's counseling process in this case was another noteworthy aspect, emphasizing shared decision-making and informed consent, which has been identified in the literature as a determinant of patient satisfaction and adherence to treatment plans [25]. The incidence of heterotopic pregnancies has risen significantly with the increased use of ART [26,27]. Compared to spontaneous pregnancies, ART-associated pregnancies carry a higher risk of EP, with reported rates ranging from 2.2% to 4.5% [28]. This increased risk is attributed to multiple ETs, technical aspects of ET, and predisposing conditions such as endometriosis [29,30].

## Conclusions

Heterotopic pregnancy, though rare, is becoming increasingly recognized as a complication of ARTs. Early and accurate diagnosis through ultrasound is crucial in reducing delays and ensuring better outcomes for both the mother and the intrauterine pregnancy. Ultrasound not only serves to confirm the presence of a heterotopic pregnancy but also aids in timely decision-making to minimize associated risks.

Key risk factors such as endometriosis, pelvic inflammatory disease, and ET techniques should be carefully assessed when planning ART procedures. Effective management requires a multidisciplinary approach, involving reproductive endocrinologists, radiologists, and surgeons, to deliver optimal care. Early detection and prompt surgical interventions, such as laparoscopic salpingectomy, are critical for preserving intrauterine pregnancy and minimizing maternal complications.

Patient counseling plays a pivotal role in these cases, ensuring informed decision-making and setting realistic expectations for outcomes. Continued research is needed to improve our understanding of long-term outcomes and to refine management strategies, particularly as ART techniques and protocols evolve. By addressing risk factors and advancing diagnostic and therapeutic approaches, the care of patients with heterotopic pregnancy can be significantly enhanced.
